# Autophagy and Senescence: The Molecular Mechanisms and Implications in Liver Diseases

**DOI:** 10.3390/ijms242316880

**Published:** 2023-11-28

**Authors:** Qiao Li, Yan Lin, Guangyu Liang, Nanyin Xiao, Heng Zhang, Xiao Yang, Jiankun Yang, Anding Liu

**Affiliations:** Experimental Medicine Center, Tongji Hospital, Tongji Medical College, Huazhong University of Sciences and Technology, Wuhan 430100, China; liqiaograce@163.com (Q.L.); ylin64367@gmail.com (Y.L.); catherinel0113@163.com (G.L.); xiaony2018@163.com (N.X.); zh61221@gmail.com (H.Z.); yx19920716@yeah.net (X.Y.); yangjiankun8023@163.com (J.Y.)

**Keywords:** autophagy, senescence, SASP, NAFLD, liver fibrosis, liver cancer

## Abstract

The liver is the primary organ accountable for complex physiological functions, including lipid metabolism, toxic chemical degradation, bile acid synthesis, and glucose metabolism. Liver function homeostasis is essential for the stability of bodily functions and is involved in the complex regulation of the balance between cell proliferation and cell death. Cell proliferation-halting mechanisms, including autophagy and senescence, are implicated in the development of several liver diseases, such as cholestasis, viral hepatitis, nonalcoholic fatty liver disease, liver fibrosis, and hepatocellular carcinoma. Among various cell death mechanisms, autophagy is a highly conserved and self-degradative cellular process that recycles damaged organelles, cellular debris, and proteins. This process also provides the substrate for further metabolism. A defect in the autophagy machinery can lead to premature diseases, accelerated aging, inflammatory state, tumorigenesis, and cellular senescence. Senescence, another cell death type, is an active player in eliminating premalignant cells. At the same time, senescent cells can affect the function of neighboring cells by secreting the senescence-associated secretory phenotype and induce paracrine senescence. Autophagy can promote and delay cellular senescence under different contexts. This review decodes the roles of autophagy and senescence in multiple liver diseases to achieve a better understanding of the regulatory mechanisms and implications of autophagy and senescence in various liver diseases.

## 1. Introduction 

The liver is the most vital organ involved in metabolism and detoxification in adults. This organ is composed of hepatocytes, Kupffer cells, hepatic stellate cells, dendric cells, liver sinusoidal endothelial cells (LSECs), and neutrophils [1]. Hepatocytes are parenchymal cells of the liver and synthesize proteins, including fibrinogen and albumin [1]. Kupffer cells are specialized macrophages residing in the liver and are classified into proinflammatory M1 macrophages and anti-inflammatory M2 macrophages [1]. LSECs are endothelial cells lining the liver sinusoids [1] and undergo complex adaptation during aging. Increased serum γ-glutamyl transferase, high-density protein, cholesterol levels, and reduced hepatic mass and blood perfusion are noteworthy characteristics of aging livers [1]. Chronic liver diseases including nonalcoholic fatty liver disease (NAFLD), chronic hepatitis, liver fibrosis, and hepatocellular carcinoma (HCC) are associated with the aging of the liver [1].

Autophagy, another type of cell death, involves degradation of cellular organelles and proteins to exclude cellular debris and sustain the homeostasis of cellular functions [2]. Except for the maintenance of proteostasis, autophagy functions in senescence regulation, host defense against pathogens, and cellular debris degradation, thereby providing energetic substrates for metabolism [2]. A dysfunctional autophagy machinery is implicated in multiple liver diseases and is crosslinked to several hallmarks of aging [2]. To gain an understanding of the underlying mechanism and implications of autophagy and senescence in multiple liver diseases, the current review will delineate the concept of the cell death processes, autophagy and senescence, and the roles of these processes in multiple liver diseases in the following sections.

## 2. Classification of Autophagy

Apoptosis, necrosis, and autophagy are three common types of programmed cell death. Autophagy is a cellular process that can eradicate metabolites and unfolded proteins from cells [3]. When autophagy accompanies cell death, it is termed type II programmed cell death [3]. Based on their different mechanisms, autophagy is classified into several types, including macro-autophagy, micro-autophagy, and chaperon-mediated autophagy (CMA) [4].

### 2.1. Macroautophagy

**Figure 1:**
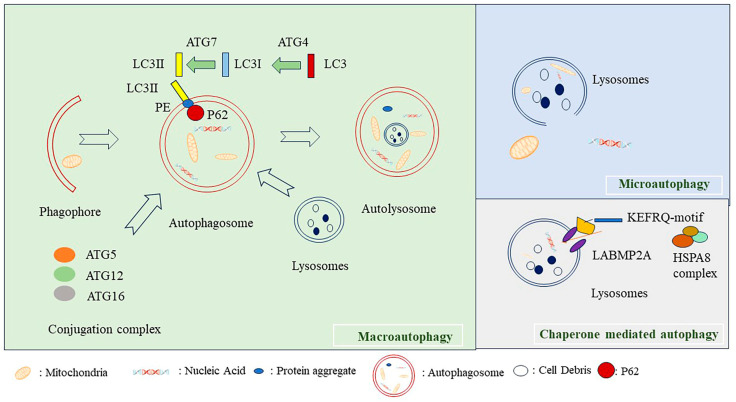
Autophagy machinery. Autophagy can be classified as macroautophagy, microautophagy, and chaperone-mediated autophagy. Macroautophagy is initiated from the phagophore that swallows the cellular debris and organelles in the cells, and then the phagophores elongate to form autophagosome. Autophagosome fuse with the lysosome and form autolysosome. LC3 is transformed to LC3I by ATG4, and, sequentially, LC3I is changed to LC3II by ATG7. P62 links the proteins with ubiquitin to the autophagosome membrane. Autophagy marker proteins LC3II insert into the autophagosome membrane. Microautophagy involves the direct digestion of cellular proteins by lysosome. In chaperone-mediated autophagy, a pentapeptide KFERQ-like motif containing substrate is recognized by the molecular chaperone heat shock cognate 71 kDa protein (HSC70), while the HSC70 complex binds with the lysosomal membrane protein LAMP2A and translocate proteins into lysosome for degradation [5].

Macroautophagy is the most common autophagy type. External stimuli, such as nutrient deprivation and oxidative stress, can activate this process. Macroautophagy originates from the endoplasmic reticulum and is characterized by the formation of a phagophore and the unique double-membraned autophagosome [4]. The autophagosome traffics the cargoes carried to fuse with lysosomes and thus form autolysosomes to degrade the selected cellular components [4] (Figure 1).

### 2.2. Microautophagy

Microautophagy can be categorized into two types: fission-type and fusion-type microautophagy [6]. Microautophagy does not involve autophagosome formation [7], but involves the protrusion or invagination of the lysosomal membrane [6]. Mammalian cells mostly undergo fission-type microautophagy [6] (Figure 1).

### 2.3. Mhaperon-Mediated Autophagy

In CMA, intracellular soluble components are directly degraded by lysosomes. The substrates of this type of autophagy must contain a particular pentapeptide KFERQ-like motif [8]. The molecular chaperone heat shock cognate (HSC70), 71 kDa protein [9], recognizes this motif and further binds with lysosomal-associated membrane protein type 2A (LAMP2A), a lysosomal membrane protein. The binding complex can translocate proteins across the lysosomal membrane for subsequent degradation [5] (Figure 1).

Other types of autophagy include mitochondrial autophagy (mitophagy), glycophagy, lipophagy, and nucleophagy [4]. Mitophagy is a quality control mechanism of mitochondria, wherein damaged mitochondria are marked with PTEN-induced kinase 1 (PINK1) and then degraded [10].

## 3. Regulatory Mechanisms of Autophagy

The core autophagy machinery involves several steps: initiation, nucleation, elongation, closure, maturation, and degradation or extrusion [11]. Autophagy is elegantly regulated. The ATG conjugation system is a key regulatory mechanism in autophagy that regulates autophagosome formation [12]. Two key steps are implicated in autophagy: (1) Atg5–Atg12 conjugation interacts with Atg16L. This is essential for autophagosome formation [13]. (2) Microtubule-associated protein 1 light chain 3 (LC3) processing and insertion. The key regulators of autophagy are summarized as follows.

### 3.1. ATG5, P62, and LC3

P62 and LC3 are classic markers of autophagy [12]. Proteins that need to be degraded through autophagy are ubiquitinated and engulfed by the phagophore, which later forms an autophagosome [14]. P62 as an autophagy adaptor protein is a ubiquitin-binding protein [14]. It binds with the ubiquitinated protein and delivers it to the autophagosome for degradation [15]. P62//sequestosome-1 binds with LC3 and degrades the protein through autophagy [15]. In the case of a defective autophagy machinery, the cellular P62 level increases [16].

The levels of LC3, a maker of autophagosomes and autolysosomes [17], is increased during autophagy [18]. LC3 is involved in recruiting autophagy cargos to autophagosomes [17]. LC3 is processed by ATG4 to form the cytosolic form LC3-I, followed by ATG7 to form the membrane-bound form LC3-II later [17]. The conversion of LC3-I to LC3-II is a prominent marker of autophagy [18] and is mediated by the Atg-5-Atg12-Atg16L1 complex [19]. When autophagy is inhibited at the early stage, LC3-I conversion to LC3-II and Beclin 1 and ATG5 levels decline [20]. Blocking of the autophagy flux during late autophagy leads to autophagosome aggregation [21]. When autophagosomes are formed normally, but lysosomal degradation is defective, cellular levels of both p62 and LC3 increase [22].

### 3.2. PI3K/Akt/mTOR Signaling in Autophagy Regulation

**Figure 2:**
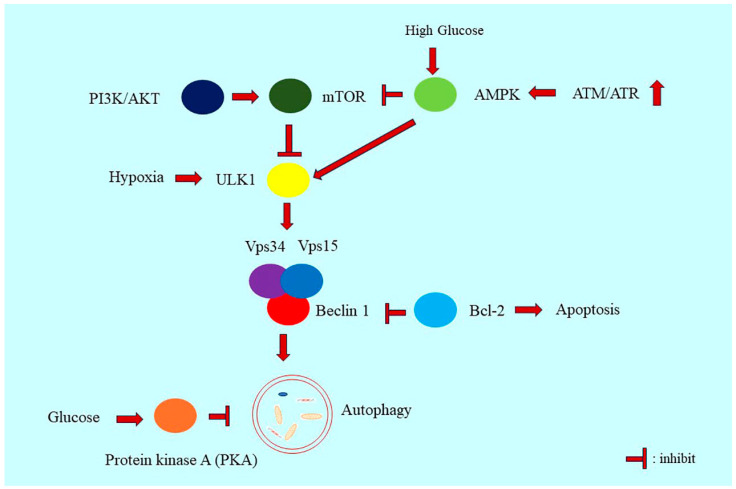
Regulation of autophagy machinery. The autophagy machinery is elegantly regulated. Autophagy can be regulated by AMPK signaling, which can sense the nutrient supplement. The increase in the AMP/ATP ratio activates AMPK signaling and initiate autophagy. AMPK negatively regulates the mTOR that inhibits autophagy. PI3K/AKT signaling phosphorylates mTOR to inhibit autophagy. Beclin 1 is s a linker from ULK1 to VPS34) complex. Beclin 1 is phosphorylated by ULK1 upon amino acid starvation, activate lipid kinase VPS34 complex and induce autophagy. “┴” represents blocking.

Class I phosphatidylinositol 3-kinase (PI3K) activates protein kinase B (Akt) and downstreams the mammalian target of rapamycin (mTOR) signaling [23]. PI3K phosphorylation catalyzes the formation of phosphatidylinositol-3,4,5-triphosphate (PIP3). PIP3 binds to Akt/protein kinase B and phosphoinositide-dependent kinase-1 (PDK1). PDK1 then phosphorylates AKT, which in turn phosphorylates mTOR to inhibit autophagy [24]. Mammalian target of rapamycin complex 1 (mTORC1) signaling negatively regulates the activity of the unc-51–like autophagy-activating kinase (ULK)1 complex [25] (Figure 2). Upon starvation, mTOR signaling is inhibited, and the autophagosome is formed with the activation of the ULK1/2 complex [25,26]. mTOR signaling can inhibit the formation of the Atg1–Atg13–Atg17 complex [27]. The mTOR inhibitor rapamycin amplifies autophagy [25]. Chloroquine can inhibit lysosomal and autolysosomal degradation, thereby inhibiting the late stage of autophagy [28].

### 3.3. AMPK Signaling in Autophagy Regulation

AMP-activated protein kinase (AMPK) and mTOR phosphorylate ULK1 prevent Ulk1 activation [25,29]. AMPK negatively regulates mTORC1 signaling activity [26] (Figure 2). During starvation, an increase in the AMP/ATP ratio activates AMPK signaling [30] and initiates autophagy [25]. Tumor suppressor p53 can also activate AMPK [31]. p53 acts like a double-edged sword in autophagy regulation [32]. Nuclear p53 activates autophagy [33], whereas cytoplasmic p53 inhibits autophagy [32]. Cytoplasmic-acetylated p53 mediates ubiquitin-mediated Beclin 1 degradation and suppresses autophagy [34]. Cytoplasmic p53 also activates AMPK/mTOR signaling and inhibits autophagy [35].

### 3.4. Beclin 1 in Autophagy Regulation

Beclin 1 can mediate autophagy by serving as a linker connecting ULK1 to the autophagy-specific vacuolar protein-sorting 34 (VPS34) complex [36]. Upon amino acid starvation or mTOR inhibition, Beclin 1 is phosphorylated by ULK1 [36], which then enhances the activity of the lipid kinase VPS34 complex and further induces autophagy [36]. The loss of Atg5, liver-specific Atg7, or Beclin 1 in mice causes the aggregation of proteins, lipid droplets, and damaged organelles in cells [37] (Figure 2).

The mammalian autophagy protein, Beclin1, acts as a bridge between autophagy and cell death [38]. Anti-apoptotic Bcl-2 family members can interact with Beclin 1, thereby leading to the inhibition of autophagy [39]. When Fernández et al. inhibited the interaction of Beclin 1 with the negative regulator BCL2 in mice with a Phe121Ala mutation in Beclin 1, the basal level of autophagic flux was increased [40]. The lifespan of the belin1 mutation mice increased, further indicating that the disruption of the Beclin 1–BCL2 complex can prolong the health span of mammals [40].

### 3.5. Extracellular Signal-Regulated Kinase Signaling

The extracellular signal-regulated kinase (ERK) signaling pathway is another regulatory signaling pathway involved in autophagy regulation [41,42]. ERK can mediate tumor necrosis factor α (TNFα)-induced p53 phosphorylation on serine 392 and further induce autophagy [43]. Mitochondrial fission can trigger high mobility group box-1 protein (HMGB1)-induced autophagy by ERK signaling [44]. GTPase dynamin-related protein 1 (Drp1) translocates from the cytoplasm to mitochondria and induces mitochondrial fission [44]. HMGB1 increases Drp1 phosphorylation and Drp1-dependent mitochondrial fission by activating the ERK1/2 signaling pathway. ERK1/2 phosphorylates Drp1 and further mediates HMGB1-induced autophagy [44]. ERK and JNK signaling pathways also contribute to transforming growth factor β1 (TGF-β1)-induced autophagy in hepatic stellate cells [45].

## 4. The Role of Autophagy in Liver Diseases

Autophagy plays profound roles in various human diseases, including cancer, neurodegeneration, genetic disorders, autoimmunity, and cardiovascular diseases. Yamamoto et al. reported that selective autophagy can induce the immune escape of pancreatic cancer [46]. In pancreatic ductal adenocarcinoma (PDAC), MHC-I were enriched in autophagosomes and lysosomes and degraded through autophagy [46]. Inhibition of autophagy can restore the MHC-I expression and CD8^+^ T cell-mediated tumor surveillance [46]. Autophagy impairment is associated with cardiac dysfunction [47,48,49]. Cardiac-specific loss of Atg5 causes autophagy impairment in cardiomyocytes and causes age-related cardiomyopathy [47,48]. Parkin protein, a E3 ubiquitin ligase, is involved in the removal of damaged mitochondria through autophagy [49]. Parkin mutation is associated with the development of Parkinson disease [50]. Kubli et al. reported that Parkin deficiency in myocytes is related to the impairment of mitophagy and causes dysfunctional mitochondria accumulation and increased oxidative stress in the myocytes after the cardio infarction [49]. HTT (huntingtin) interact with ULK1 and SQSTM1/p62 and with mitophagy components [51]. HTT protein mutation causes the impaired removal of dysfunctional mitochondria by mitophagy, and is related with the development of Huntington disease (HD) [51]. Impairment of autophagy is also involved in the progression of ankylosing spondylitis [52], autosomal dominant inherited sensorimotor neuropathy Charcot-Marie-Tooth disease type 2A (CMT2A) [53], neurodegenerative diseases Amytrophic Lateral Sclerosis (ALS), Alzheimer’s disease (AD) [54], Leber’s hereditary optic neuropathy [55], myocardial ischemia/reperfusion injury induced autosis [56], cystic fibrosis [57], Myocardial Ischemia [58], and tumorigenesis [59,60].

Autophagy provides glucose, amino acids, and free fatty acids to hepatocytes and sustains liver function homeostasis [37]. Under nutrient starvation and external stress conditions, autophagy particularly provides substrates and materials for metabolism [61]. Lipids degraded during autophagy produce free fatty acids, which serve as substrates for β-oxidation and the tricarboxylic acid (TCA) cycle [62].

When age increases, autophagy activity decreases [63]. Deterioration of autophagy increases the risk of liver diseases [64] and carcinogenesis [65].

### 4.1. The Role of Autophagy in Liver Fibrosis, NAFLD and NASH

Liver fibrosis as a wound-healing response contributed to the development of chronic liver dysfunction. Autophagy plays different roles in the hepatocytes, LSECs, and hepatic stellate cells.

NAFLD is a metabolic disorder and a main cause of cirrhosis [66]. Activation of hepatic autophagy alleviate liver steatosis [66]. Accumulation of p62 and LC3II was observed in steatosis and NASH patients, which indicates the decreased autophagy in hepatic steatosis and NASH [67]. The impaired autophagic flux in NAFLD increases the ER stress leading to apoptosis of hepatocyte [67]. Autophagy dysfunction is associated with mitochondrial oxidative stress and ER stress, eventually causing insulin resistance [68]. Insulin resistance is the main mechanism of NAFLD development [69].

The development of NASH is characterized with steatosis, inflammation, and a certain degree of fibrosis [70]. Autophagy is involved in the progression of liver steatosis to NASH [71]. The autophagy machinery is defective in the LSECs of nonalcoholic steatohepatitis (NASH) patients, and this defect favors liver fibrosis development [72]. The autophagy dysfunction boosts the upregulation of inflammatory pathways including CCL2, CCL5, IL-6, and VCAM-1 expression [72]. Deficient autophagy in the liver endothelial cells of NASH patients enhances the inflammatory feature and the expression of endothelial-to-mesenchymal factors in the early stages of NASH [72]. SREBP cleavage-activating proteins (SCAP) are also involved in the pathogenesis of NASH [73]. Sterol regulatory element-binding proteins (SREBPs) are important lipogenesis regulators. SCAP escorts the SREBP to the Golgi apparatus for lipid biosynthesis. Deletion of SCAP in the PTEN^ΔL^ mice activates the Akt–mTOR pathway which inhibits autophagy and induces liver injury, hepatic steatosis, and carcinogenesis [73].

DAMPs, e.g., HMGB1, link cell death mechanism autophagy with the onset of liver diseases, e.g., liver fibrosis [74]. HMGB1 is released by hepatocytes after acute liver failure [74]. HMGB1 can induce autophagy and activate HSCs [75]. HMGB1 is associated with NAFLD, alcoholic liver disease (ALD), liver I/R, hepatocyte carcinoma, and liver fibrosis [76,77]. In autophagy-deficient livers, HMGB1 promotes tumorigenesis [78]. NRF2 activation causes HMGB1 release by the participation of inflammasomes [78].

In hepatocytes, autophagy maintains cellular homeostasis by removing damaged mitochondria and accumulated fat [64,79]. A steatotic liver with autophagy dysfunction is prone to develop fibrosis [66]. In activated HSCs, autophagy promotes the development of fibrosis by getting rid of cytoplasmic lipid droplets and activating HSCs [80]. The autophagy level in ISECs is reduced in mice with liver fibrosis [81]. The autophagy impairment in ISECs will increase the oxygen stress and reduce the nitric oxide level, which is important for limiting HSCs activation and extracellular matrix production [81]. Impaired autophagy efflux in liver endothelial cells aggravates the deterioration of the liver function in liver fibrosis [82], and this is associated with the insufficient antioxidant response of LSECs [82]. 

### 4.2. Autophagy and Liver Malignancies

HCC is the most common type of liver cancer [83], and autophagy plays a paradoxical role in HCC [84]. The role of autophagy in HCC changes with the progression of hepatocytic tumor cells [85]. Under certain circumstances, autophagy functions as a tumor suppressor [84]. At the early tumorigenesis stage, autophagy maintains liver homeostasis by excluding premalignant liver cells and degrading damaged organelles and protein aggregates through lysosomes [84]. In Beclin 1 heterozygous-disrupted mice, autophagy activity was inhibited, which consequently increased the development frequency of spontaneous malignancies, along with an increase in the risk of hepatitis B virus-induced premalignancies [86]. This study offered convincing evidence that autophagy can inhibit tumorigenesis and the autophagy-related gene Beclin 1 functions as a tumor suppressor gene [86]. Mathew et al. investigated the consistent tumor-suppressive function of Beclin 1 in order to limit chromosomal instability [87]. The authors illustrated that Beclin 1 loss promotes tumor development [87]. Beclin 1 loss increases genomic instability, including DNA damage and gene amplification, caused by the impairment of autophagy and promotes tumorigenesis [87]. The expression of autophagic genes Beclin 1 is decreased in the HCC tumor samples, comparing to the adjacent normal tissues. In antiapoptotic protein Bcl-xL+ samples, autophagic level is lower. A correlation between poorer patients’ prognosis and absence of Beclin 1 expression only present in the Bcl-xL+ patients group [88]. Aberrant accumulation of P62, which promotes tumorigenesis, was observed in different cancers [89]. Autophagy can alleviate P62 accumulation and inhibit tumorigenesis [89]. Autophagy enhancement primes the macrophage to M1 polarization [66].

In the late stage of tumor development, autophagy can be tumor-promoting in established tumors [89]. Autophagy can favor tumorigenesis, metastasis, and resistance to chemotherapy [89]. Autophagy promotes chemoresistance in liver cancer cells [90] (Table 1).

### 4.3. Autophagy and Other Liver Dysfunction

Ischemia–reperfusion injury (IRI) is damage caused during the ischemic period due to the lack of oxygen and reperfusion to organs [91]. IRI leads to liver transplantation-associated morbidity [92]. The chief mechanism of damage in IRI is activation of pro-inflammatory pathways and oxidative stress in ischemic cells [93]. Cell death including autophagy, apoptosis, and necrosis can be activated during IRI [93].

Autophagy can have a protective role in IRI [94]. Because hepatic autophagy is defective in liver steatosis, steatotic livers are more vulnerable to IRI [94]. Ischemic preconditioning can attenuate IRI by activating heme oxygenase-1 signaling [94]. Autophagy can be defective during IRI [92]. According to Xi et al., a member of the Nod-like receptor (NLR) family, nucleotide-binding oligomerization domain protein 1 (NOD1), can activate autophagy and aggravate hepatic IRI [92]. Atg5 mediates this effect after NOD1 activation [92].

Growth differentiation factor 11 (GDF11), a member of the TGF-β superfamily, significantly activates mTORC1 and deteriorates liver regeneration in mice undergoing partial hepatectomy [95]. By activating the TGF-β-SMAD2/3 signaling pathway, GDF11 suppresses the cell cycle progression of liver cells [96], consequently impairing liver regeneration following liver IRI [96]. Treatment of mice with young plasma activates age-impaired autophagy [97]. The plasma treatment increases AMPK phosphorylation and activates ULK1 and autophagy to ameliorate age-dependent liver IRI [97].

IRI is a sterile inflammation process, and damage-associated molecular patterns (DAMPs), such as HMGB1 and histones, are released during liver IRI. DAMPs activate Toll-like receptors (TLR) 4 and 9 and exacerbate hepatic injury [98]. DAMPs also induce the formation of neutrophil extracellular traps (NET) during IRI [99]. Peptidyl arginine deiminase 4 inhibitor or DNase I-mediated inhibition of NET formation alleviates histone-mediated liver IRI [99].

## 5. Senescence and Senescence-Associated Biomarkers

Senescence is a permanent halt of the cell proliferation cycle and is induced by internal or external insults [100]. DNA damage, telomere attrition, and mitochondrial dysfunction can trigger the activation of cellular senescence [100]. The senescent cells secrete a group of cytokines with autocrine, paracrine, and endocrine activities, termed as senescence-associated secretory phenotypes (SASP) [101]. Senescence-associated beta-galactosidase activity (SA-βgal) and SASP are two classical characteristics of senescence [102]. Lamin B1 and α-fucosidase have been used as senescence-associated biomarkers.

Senescence-associated heterochromatin foci (SAHF) is another biomarker of senescence. The formed SAHF accounts for the condensed chromatin structure, thereby sequestering cell proliferation-related genes and contributing to senescence [103].

## 6. Classification of Senescence

**Figure 3:**
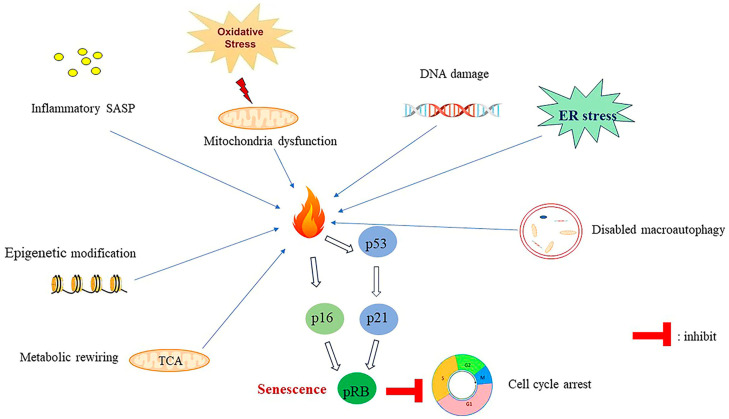
Inducers of senescence. Senescence can be triggered by multiple inducers. Senescent cells can secret SASP that includes inflammatory cytokines to trigger the senescence of the neighboring cells. Oxidative stress induced by various stimulus can cause mitochondria dysfunction and induce senescence. DNA damage can also activate p53 signaling and induce senescence. ER stress induced by protein aggregates can induce senescence. Defective macroautophagy, epigenetic rewiring, and metabolism dysregulation induce senescence via multiple mechanisms. Diverse inducers activate senescence through the activation of p53 and p16^Ink4A^ signaling, which further regulates the pRB and results in the arrest of the cell cycle progression and, eventually, senescence. “┴” represents blocking.

Senescence is categorized into three major types: oncogene-induced senescence, which is induced through the activation of oncogenes (e.g., ras and myc); replicative senescence caused by the progressive attrition of telomeres; and stress-induced senescence, which is induced by stressors such as chemotherapy and radiotherapy burns [104,105] (Figure 3).

### 6.1. Replicative Senescence 

Telomere is a conserved structure presented at the chromosome’s end and is involved in maintaining genome stability. Semiconservative DNA replication causes the linear chromosome to eventually shorten. Telomerase is a DNA-dependent DNA polymerase extending the 3′ ends of chromosomes [106]. This enzyme is composed of an RNA template component, that is, human telomerase RNA, and human telomeric reverse transcriptase [107]. Telomerase is silent in most somatic cells [107,108]. During replication, once the cells reach the “Hayflick limitation” [109], which is a phenomenon wherein normal human cells reach the limitation established for division, the cell cycle regulator p21CIP1 and p16^INK4a^ further inhibit the activation of cyclin-dependent kinases CDK4, CDK6, and CDK2 [110], leading to the phosphorylation of the retinoblastoma protein (pRb), which halts cell cycle progression and induces replicative senescence in the cells [110].

### 6.2. Oncogene and Stress-Induced Senescence

Oncogene activation, and chemotherapy or radiotherapy can also activate senescence [110]. The damage to DNA or a stress signal activates p53/p21^CIP1^ and p16^INK4a^/RB signaling [111] and inhibits CDK4, CDK6, and CDK2, thereby inducing cell proliferation arrest [111].

## 7. Mechanisms of Senescence Induction

### 7.1. SASP-Induced Senescence

SASP can induce the evasion of immune surveillance and tissue inflammation [110]. Inflammatory SASP can elicit chronic systemic inflammation and drive senescence [110]. The senescent cells affect neighbor cells and remodel the microenvironment by secreting robust SASP [112]. These cells secrete cytokines and chemokines, including TGF-β family members and vascular endothelial growth factor (VEGF), to induce senescence in neighboring cells, which is termed paracrine senescence [113]. Released SASP can cause chronic inflammation and tissue dysfunction, which contribute significantly to aging [114]. SASP, including IL-8, IL-6, and insulin-like growth factor-binding protein 7 (IGFBP7), promote senescence [115].

### 7.2. Epigenetic Regulation-Induced Senescence

DNA methylation, chromatin remodeling, and RNA modification are epigenetic alterations that commonly occur in senescent cells [116,117]. These cells present characteristic epigenetic markers and exhibit chromatin remodeling [116], with acetylation and methylation being the most common epigenetic modification machineries of senescence [116]. In replicative senescent cells, methylation levels of H4K16Ac, H3K4me3, H3K9me3, and H3K27me3 decrease [118]. Senescent cells can form SAHF [119]. SAHF can recruit the retinoblastoma tumor suppressor to E2F-responsive promoters and inhibit the transcription of E2F target genes [103]. These foci also recruit DNA methyltransferases to regulate DNA hypermethylation [118].

Deacetylation of various substrates mediated by Sirtuin (SIRT) family members is deeply involved in senescence regulation [120,121]. SIRTs, as part of the Na (+)/dicarboxylate co-transporter (NADC)-dependent histone deacetylase III class of enzymes, have become key regulators of senescence [120]. SIRT1 expression by endothelial cells decreases with aging [120]. In replicative and oncogene-induced senescence, SIRT2 expression increases, and this increase is dependent on the p53 status, with p53-binding sites present on the SIRT2 promoter [121].

RNA modification is another epigenetic regulation mechanism of senescence [122]. N6-methyladenosine (m6A) is one of the crucial mRNA modification mechanisms [122]. RNA methyltransferase, RNA demethylase, and m6A-binding proteins collectively regulate the m6A RNA methylation level [123]. RNA methyltransferase-like protein 3 (METTL3) is a key RNA methyltransferase involved in regulating m6A RNA methylation levels [124]. m6A methylation was recently found to be involved in senescence regulation [122]. METTL3 deficiency contributes to the senescence of skeletal muscles found by m6A profiles [125]. METTL3 modifies nephronectin (NPNT) mRNA levels through m6A RNA methylation. Insulin-like growth factor 2 mRNA-binding protein 1 (IGF2BP1) can bind and stabilize NPNT mRNA, thus reducing the development of myotube senescence [125].

### 7.3. Deregulation of Cellular Metabolism Induce Senescence

Senescence is associated with metabolism disorder and mitochondrial dysfunction. Cellular senescence has a causative role in metabolic diseases, including type 2 diabetes (T2D), NAFLD, and obesity [126].

The role of adipose tissues, including white adipose tissue (WAT) and brown adipose tissue (BAT), in aging has been recognized [127]. WAT is the earliest organ that undergoes aging [128]. Adipose tissue secrets the proinflammatory cytokines IL-1, IL-6, and TNF-α and contributes to an inflammatory state [127]. Adipose tissue-derived SASP, including IL-6, IL-8, and IGFBP7, accelerate aging [127].

Glycolysis, TCA cycle, and fatty acid β-oxidation are pivotal metabolic mechanisms of intracellular metabolism [129]. Glycolysis serves as the major energy source under limited oxygen conditions [129]. Reprogramming of metabolism is characteristic in senescent cells [130]. These cells are prone to glycolysis. Hypoxia increases DNA damage and p53 activation, which further increase the glycolysis level in lung epithelial cells [131]. During oncogene-induced senescence, genotoxic stress-induced senescence, and replicative senescence, the glycolysis level is increased [132]. TCA cycle upregulation occurs in all of the aforementioned three types of senescence [133].

## 8. Implications of Senescence in Liver Diseases

Senescence occurs in various human organs, and multiple chronic disorders are related to cellular senescence, such as T2D, NAFLD, cancer, cardiovascular disease, and obesity [126]. In liver diseases, senescence also plays a profound role.

### 8.1. The Role of Cellular Senescence in Liver Cancer

**Figure 4:**
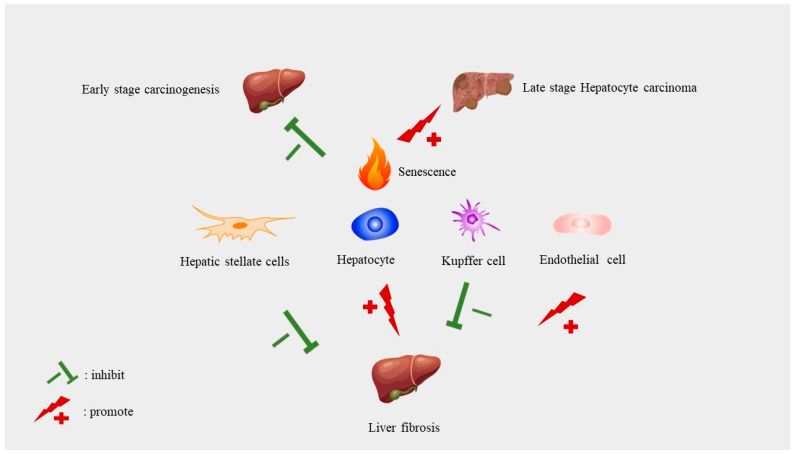
Liver cell senescence in liver fibrosis and carcinogenesis. The senescence of different cell types has a complex effect on the progression of liver diseases. Senescent human hepatic stellate cells ameliorate the development of liver fibrosis. In fact, the senescence of hepatocyte accelerates the progression of liver fibrosis. Liver sinusoidal endothelial cell (LSEC) senescence aggregates liver fibrosis as well. In liver carcinogenesis, at the early stage of hepatocyte carcinoma, senescence eliminates the premalignant liver cells and prevents the carcinogenesis of liver cancer. At the late stage of hepatocyte carcinoma, once the liver tumor is established, senescence can exacerbate tumor development.

Senescence regulates multiple hallmarks of cancer through diverse mechanisms. It can be tumor-promoting and tumor-suppressive under different circumstances [65]. Senescence can suppress tumors by stopping the cells with genomic defects from entering the cell cycle and terminates the expansion of premalignant cells [134]. In the late stage of aging, senescence can promote tumor growth and development [65]. This has been summarized by George Williams in 1957 as the “antagonistic pleiotropy hypothesis” of aging [135] (Figure 4).

In the early stage of tumor development, senescence can inhibit tumorigenesis of liver cancer cells by eradicating premalignant hepatocytes [136]. Monocytes/macrophages are necessary for the surveillance of premalignant hepatocytes by CD4^+^ T cells [136]. The surveillance failure of premalignant senescent hepatocytes promotes the aggravation of murine HCCs [136]. The p53 mutation, which causes senescence abrogation, is HCC development promotive [137]. p53 restoration rescues the surveillance of senescence cells by immune cells [138]. Senescence of HSC is tumor promotive [139]. Gluconeogenic enzyme fructose 1,6-bisphosphatase 1 (FBP1) loss causes the aberrant lipid metabolism of hepatocytes and ER stress in hepatocyte [139]. FBP1 loss also causes senescence of HSC [139]. Senescent HSC promotes tumor development by secreting SASP, especially IL6 and CXCL1 [139].

SASP has both antitumor and protumor roles in malignant transformation [138,140]. It can promote immune surveillance against damaged cells, but excessive SASP also leads to immunosuppression, thereby benefiting tumor cells [140]. SASP contributes to the establishment of an immunosuppressive microenvironment and thus facilitates tumorigenesis [141]. Ruhland et al. reported that senescent stromal cells can promote squamous cell carcinoma tumorigenesis by modeling the tumor microenvironment to the immunosuppressive phenotype [141]. SASP IL-6 was found to orchestrate myeloid cells to inhibit antitumor T cell responses [141]. In addition, persistent SASP activation can promote malignant cell proliferation [140,142].

Numerous studies have suggested the role of SASP in HCC carcinogenesis [143]. In obesity patients, deoxycholic acid (DCA) levels in the circulation increase because of obesity-induced alterations in gut microbiota [143]. The released DCA can induce SASP of HSCs and aggravate liver cell tumorigenesis [143].

SASP induces epithelial-to-mesenchymal transition (EMT), a critical mechanism mediating tumor metastasis [144]. Senescent fibroblasts secrete SASP, which then promotes tumor progression by inducing the EMT of epithelial cells [144]. Senescent cells secrete matrix metalloproteinase (MMP)-2, MMP-3, and uPA and its regulator (PAI1) into the surroundings and induce the migration and metastasis of neighboring cells by degrading the ECM and invading the basement membrane [144]. Thus, senescent cells can secrete SASP to promote tumor metastasis [144]. On the other hand, SASP can be tumor-suppressive by recruiting and activating immune cells. SASP recruits and activates CD4^+^ and CD8^+^ T cells to exclude premalignant liver cells and inhibit tumorigenesis [145]. The complex role of SASP in cancer needs to be investigated under specific conditions.

### 8.2. Senescence in Liver Fibrosis and Cirrhosis

Liver fibrosis is a pathological state occurring before exacerbation into liver cirrhosis. The contribution of senescence of different cell types as part of liver fibrosis is different. The senescence of liver hepatic stellate cells reduces liver fibrosis by reducing the secretion of ECM components and countering liver fibrosis development [124]. The senescent liver hepatic stellate cells increase the secretion of ECM-degrading enzymes [146]. Insulin-like growth factors I (IGF1), IL-10, and IL-22 can induce HSC senescence [147].

LSEC remodeling is involved in liver fibrosis [148]. Senescence and inflammation were detected in the LSECs of aged mouse models [149]. Upregulation of CDKN2A (p16) and γ-H2AX and downregulation of lamin B1 were observed in aged LSECs [150]. Shear stress of blood flow can also induce LSEC senescence [148]. LSEC senescence contributes to liver fibrosis development [148]. VEGF, which can regulate sinusoidal permeability, benefits fibrosis resolution and repair in mice [151] (Figure 4).

Senescence of hepatocytes contributes to liver fibrosis development [146]. Conditioned medium from senescent HepG2 hepatocyte cells activates human hepatic stellate cells and significantly upregulates the expression of inflammatory and fibrogenic genes [152]. SASP contained in the senescent hepatocyte-conditioned media regulates the gene expression profile of the HSCs and promotes the fibrosis of the HSCs [152] This study provided a causal connection between hepatocytes and liver fibrosis [152]. Yu et al. reported that hepatocyte senescence induced by lipid deposition in hepatocytes activates HSC through the nuclear factor erythroid 2-related factor 2 (Nrf2)-antioxidant response element pathway [153].

Liver cirrhosis, as the end result of fibrosis, is a common disease occurring after a long period of infection with hepatitis B and C viruses or alcohol assumption and other aetiologies [15]. The late stage of liver cirrhosis is associated with a high risk of liver cancer [83]. Liver cirrhosis development is related to hepatocyte senescence [154]. According to Wiemann et al., hepatocyte senescence is a marker of human liver cirrhosis [154]. Natural killer cells can reduce liver cirrhosis progression by removing senescent cells [124] (Table 2). 

### 8.3. Senescence and NAFLD

Liver senescence is associated with divergent liver diseases, such as NAFLD and HCC [1]. It is predicted that NAFLD might become a leading cause of mortality in end-stage liver diseases [155]. Metabolic dysregulation, including obesity, contributes to NAFLD development [156]. NAFLD is characterized by steatosis and triglyceride accumulation in hepatocytes [156]. Obesity increases ROS production and accelerates cellular senescence [156]. Ogrodnik et al. reported that mice fed with Special Diet Services ad libitum develop fatty liver and accumulation of senescent hepatocytes [157].

In aged livers, the expression of p53, p21, and p16Ink4A and loss of LMNB1 are increased [150]. Aged livers also exhibit shortening of telomere length [150]. In the liver of NAFLD/NASH patients, the expression of senescence markers, such as SAβgal activity, γ-H2AX activity, and proinflammatory SASP, are upregulated [150]. Park et al. reported that the expression of the senescence marker protein-30 (SMP30) is lower in the liver tissue of NAFLD patients. SMP30 might be involved in the pathogenesis of NAFLD by functioning as an antiapoptotic protein and antioxidant [158].Serine/cysteine proteinase inhibitors (SERPINs), candidate biomarkers of cellular senescence, can remodel the ECM [159]. The hypoxia-inducible factor-2α-dependent cysteine protease inhibitor, SerpinB3, is upregulated in NAFLD [160,161]. Insulin signaling resistance indicates the onset of an aging liver [162], which then leads to alteration of hepatic lipid metabolism. Genes involved in insulin signaling, such as IGF1 and IGFBP2, are methylated in NAFLD, and methylation is reversed after bariatric surgery [163]. Excessive lipid accumulation in the liver accelerates NAFLD emergence, and at the same time, favors inflammation and induces endoplasmic reticulum stress [162], which are potential drivers of cellular senescence.

Hepatocyte senescence affects the pathogenesis of NAFLD and its progression to NASH, and further HCC, through several mechanisms. And liver senescence can be a consequence of NAFLD. Hepatic senescence functions as a driver of NAFLD development [161]. Senescent NAFLD cells exhibit aberrant oxygen stress, excessive proinflammatory cytokines, and mitochondrial dysfunction, which reinforce NAFLD development [161]. 

The aging of the immune system is associated with NASH [164].The occurrence of immunosenescence, which is the aging of the immune system [165], functions as one of the crucial regulators and as a consequence of aging [165]. Aging immune cells undergo profound modification [166], including a reduction in the immune repertoire and naive cells, with an increase in the proportion of memory cells [167]. Pattern recognition and signaling are impaired and the functions of dendritic cells are compromised during aging [168]. Immunosenescence is related to the development of NAFLD and NASH, including liver fibrosis and cirrhosis [164]. Senescent T cells secret TNF-α and IFN-γ proinflammatory cytokines [164] which mediate the crosstalk between immunosenescence and metabolic diseases [164]. Sim et al. found that hepatic T-cell senescence is a driver of insulin resistance and is associated with the development of liver fibrosis [164].

## 9. The Relationship between Autophagy and Senescence

Autophagy and senescence are two cell death forms that are implicated in the pathogenesis of multiple liver diseases. These cell death types can act in concert in liver diseases. In liver cancer, autophagy and senescence of hepatocytes occur simultaneously and interact with each other. A deficient autophagy accelerates senescence and promotes tumor progression in the liver [169]. Manipulating autophagy can prevent liver aging [170]. Modulation of the balance of autophagy and senescence in the liver delays liver aging. Inhibition of autophagy may lead to the progression of the senescent phenotype [171]; this is associated with the development of chronic liver diseases. Impairment of autophagy accompanies the progression of steatosis to NASH. Autophagy impairment is correlated with lipid accumulation and the reduced elimination of damaged organelles and proteins, impact aging related NAFLD [172].

The interaction of hepatocytes and stromal cells including HSCs, ISEC, and Kupffer cells contribute collectively to the development of liver diseases [173]. Cell death signals are important mechanisms in regulating liver inflammation [74]. Both senescence and autophagy dysregulation participate in the development of liver fibrosis [74]. Liver fibrosis is an important pathology change in liver diseases; it is essential as an aberrant wound-healing response [174]. It is related to the activation of myofibroblasts in the liver [174]. The main sources of myofibroblasts are HSCs and fibroblasts [175]. Activation of HSCs is an important mechanism contributing to the development of liver fibrosis. Senescent of HSCs can alleviate the progression of liver fibrosis [124]. On the other hand, autophagy can promote the development of liver fibrosis by providing metabolic substrates to HSCs by degrading lipid droplets [11,80]. HSCs can be activated by HMGB1 which induces autophagy and is implicated in the development of liver fibrosis [75].

A complex interaction connects autophagy and senescence. Autophagy can both activate and inhibit senescence [176]. On one hand, the accumulation of damaged proteins and organelles in the body accelerates senescence [2]. Sirtuin deacetylase activity and NAD^+^ downregulation also cause the hyperacetylation of autophagy proteins [177]. SIRT1 is a protein connecting autophagy and senescence [178]. NAD-dependent deacetylase SIRT1 activates autophagy, deacetylates NF-κB, and suppresses cellular senescence of human dermal fibroblasts [178]. SIRT1 forms a molecular complex and deacetylates autophagy proteins (Atg5, 7, and 8) [179]. SIRT1 deletion inhibits autophagy and causes damaged organelle accumulation and perinatal mortality in vivo [179]. During senescence, SIRT1 is degraded through autophagy as the nuclear autophagy substrates [180]. SIRT1 degradation through the autophagy–lysosome pathway promotes SIRT1 protein homeostasis [180]. Compared to young mice, olde mice exposed to Ischemia/reperfusion (I/R) injury show SIRT1 comprehensive loss and defective autophagy, leading to I/R injury sensitivity [181].

Autophagy also promotes cellular senescence [105]. It can degrade the nuclear lamina protein LAMINB1 (lamin B1) upon oncogenic insults through nucleus-to-cytoplasm transport, similar to SIRT1 degradation, and is related to oncogene-induced senescence [182]. The transcription factor nuclear factor erythroid 2-related factor 2 (Nrf2) is a critical factor involved in the regulation of cellular ROS stress [169]. Autophagy deficiency in the liver can activate Nrf2, which further regulates liver senescence [169]. CCL2 family chemokines promote senescence in autophagy-deficient livers [169]. CCR2-mediated inflammation promotes tumor progression in autophagy-deficient livers which lack the metabolic support provided by autophagy [169].

Partial hepatectomy (PHx) reduces β-oxidation in hepatocytes [183]. In liver-specific autophagy-related gene 5 (Atg5) knockout (KO) mice [183], PHx induced increased p62 and ubiquitinated proteins accumulation [183]. This indicates the impairment of autophagy [183]. Hepatocyte senescence indicated by SA-β-gal staining was observed in the Atg5 KO mice that had undergone PHx [183]. The study supported that autophagy in hepatocytes maintain mitochondrial homeostasis and sustain hepatocyte proliferation by preventing hepatocytic senescence [183].

AMPK signaling sustains ATP levels by increasing catabolism through autophagy and fatty acid oxidation [105]. Upregulating p53 and p21 activity promotes chronic AMPK activation in senescence, thereby reducing the NAD^+^/NADH ratio induced by mitochondrial dysfunction [105]. Insulin–IGF1–serine/threonine-protein kinase mTOR signaling is activated by higher acetyl CoA levels in aging-related autophagy inhibition [184]. Depletion of the mTOR pathway in adipocyte exacerbates liver injuries induced by alcohol gavage and affects alcohol-associated liver disease (ALD) through liver-adipose tissue crosstalk [185].

Apoptosis signaling is also crosslinked to senescence [186]. Mitochondrial membrane permeability and cytochrome C release are decisive signals during apoptosis. Apoptosis is regulated by Bcl-2 anti- and pro-apoptotic family proteins [186]. The anti-apoptosis protein Bcl-2 is upregulated during senescence, which thus links apoptosis with senescence [186]. Senescent cell anti-apoptotic pathways (SCAPs) are upregulated during senescence [187]. PI3K/AKT and p53/p21/serpine pathways are among the IGF-1-activated SCAPs, which can lead to the accumulation of senescent cells [187]. Drugs targeting SCAPs eliminate senescent cells. For example, senolytics induce the elimination of senescent cells (senolysis) [188].

The level of the CMA receptor lysosomal-associated membrane protein 2A (LAMP2A) decreases with aging, thereby leading to the impairment of chaperone-mediated autophagy [62]. CMA dysfunction increases DNA damage, which further reinforces cellular senescence [62]. CMA dysfunction is related with cellular senescence by accumulation of DNA damage and DDR activation [62]. γH2A.X, a DNA repair factor assembly regulator, is increased by reducing LAMP-2A, and induces cellular senescence [62]. CMA dysfunction is associated with the development of NAFLD [189]. CMA dysfunction is implicated in aging [190]. In senescent fibroblast, the level of CMA decreases and the levels of LAMP-2A reduced account for the impaired CMA during aging [191]. CMA dysfunction can cause lipid accumulation and induces senescence [62]. CMA degrades perlepin 2 (PLIN2) and perlepin 3 (PLIN3) [62] and allows for the degradation of lipid droplets by autophagy [62]. The impairment of CMA leads to triacylglycerols accumulation and cellular senescence [62].

GDF11 can exacerbate liver senescence by inhibiting autophagy [192]. The interaction between senescence and autophagy controls hepatic inflammation, which is related to liver carcinogenesis, fibrosis, and ischemia-reperfusion injury, NAFLD, and NASH [74]. Fibroblast growth factor 21 (FGF21) is another regulator connecting autophagy and senescence in the pathogenesis of liver diseases. FGF21, which is secreted by the liver, regulates lipid degradation through hepatic autophagy [193]. At the same time, FGF21 exhibits anti-inflammation and anti-oxidant stress functions and is protective against hepatocyte senescence [194]. Through regulating the polarization and secretory phenotypes of macrophages, FGF21 alleviates IL-6-induced hepatocyte senescence [194].

The above regulators, including GDF11 and FGF21, chaperone mediated autophagy (CMA), SIRT1, Nrf2, mTOR pathway-connect autophagy, senescence, and liver diseases. Mitochondrial function is a key mechanism linking senescence and autophagy. Defective autophagy leads to the activation of reactive oxygen species (ROS) in the mitochondria and the release of mitochondrial DNA (mtDNA) further induces cellular senescence [195]. Increased aging causes mitochondria function impairment, as well as metabolism disorder, impaired autophagy, and an increase in the incidence of multiple liver diseases. Targeting autophagy in in the pathogenesis of liver diseases in the context of ageing is a potential therapeutic strategy.

## 10. Perspectives and Conclusions

Population aging is emerging as an economic- and healthcare-related problem for the whole society. Aging-induced chronic liver diseases represent a crucial category in aging-related diseases [1]. When exploring the mechanisms of liver disease, the role of autophagy and senescence must be considered collectively. Uncovering the relationships between autophagy and senescence and that of these two cell death types with liver diseases and dissecting the underlying mechanism will help in developing therapeutic strategies for confronting liver aging and chronic liver diseases.

### Therapeutic Strategies for Liver Diseases Targeting Autophagy Dysregulation or Senescence

Understanding the underlying molecular mechanisms of chronic liver diseases contributes to the development of therapeutic strategies [173]. Autophagy is one of the key mechanisms regulating liver physiology and pathology [11]. Autophagy dysfunction has shown its potential as a therapeutic target in metabolic-associated fatty liver disease (MAFLD) [173]. Induction of autophagy through thyroid hormone, irisin, and melatonin has been found to ameliorate the development of MAFLD [173]. Autophagy can promote the development of liver fibrosis by activating HSCs [80]. Targeting autophagy in the fibrotic liver can ameliorate the development of liver fibrosis [174]. Pharmacologically inhibiting autophagy attenuates fibrogenic activity of HSCs [80]. Metformin, as an antiaging therapy, delays senescence [196]. Bharath et al. reported that metformin can ameliorate aging-related inflammation by increasing autophagy [197]. The decrease in the autophagy level cements senescence [197]. Mitochondrial autophagy that is comprised during aging can impair mitochondrial function [197]. Metformin can normalize mitochondrial dysfunction to correct the redox imbalance caused by Th17 cytokine production by CD4^+^ T cells and improves autophagy [197].

As a form of cell death, senescence induction can be beneficial in the chemotherapy of liver malignancies [198]. The induced senescent HCCs can secret SASPs which recruit tumor-clearing immune cells including CD4+ T cells, CD8+ T cells, and natural killer cells [198]. Restoration of p53 expression in murine liver carcinoma can induce cellular senescence and promote liver tumor regression [138]. Given the crucial roles of senescence in liver disease, targeting senescence pharmaceutically can be beneficial, e.g., through the application of Metformin [199]. Metformin can ameliorate the aging process of ISEC by increasing fenestration through activating AMPK and endothelial nitric oxide pathways [200]. Metformin exhibits beneficial effects in different liver diseases, e.g., NAFLD [199]. In aged septic mice, metformin can alleviate the inflammatory response by AMPK activation and PGC1α upregulation [201].

Senolytics which clear the senescent cells are emerging as novel therapeutic strategies targeting senescence [202]. Senolytics (dasatinib and quercetin, D + Q) reduced cell senescence and inhibited hepatocellular carcinoma in Cu/Zn-superoxide dismutase knockout (Sod1KO) mice [203].

In conclusion, the complex regulation of cell death mechanisms in liver diseases provides the molecular basis for the therapeutic strategies redeeming liver function and hemostasis. Delineating the mechanisms and implications of autophagy and senescence in liver diseases will potentially promote liver health amelioration, particularly in old age.

## Figures and Tables

**Table 1:** The role of autophagy in liver diseases.

Cell Type	Disease Type	Function	Reference
Hepatocyte	Fibrosis	Protect the hepatocyte from fibrosis	[64,79]
LSECs	Fibrosis	Limit the development of fibrosis	[81,82]
HSCs	Fibrosis	Accelerate the development of fibrosis	[80]
LSEC	NASH	Prevent the development of NASH	[72]
Hepatocyte	Early stage of liver cancer	Tumor-Preventing	[84]
Hepatocyte	Late stage of liver cancer	Tumor-Promoting	[89]

**Table 2:** The role of senescence in liver diseases.

Cell Type	Disease Type	Function	Reference
Hepatocyte	Fibrosis	Promoting fibrosis	[146]
LSECs	Fibrosis	Accelerating the development of fibrosis	[148]
HSCs	Fibrosis	Reducing the development of fibrosis	[124]
Hepatocyte	Cirrhosis	Contributing to liver cirrhosis	[154]
Hepatocyte	Early stage of liver cancer	Tumor-Preventing	[134]
Hepatocyte	Late stage of liver cancer	Tumor-Promoting	[65]

## Data Availability

Not applicable.
